# Reducing Crowding in Emergency Departments With Early Prediction of Hospital Admission of Adult Patients Using Biomarkers Collected at Triage: Retrospective Cohort Study

**DOI:** 10.2196/38845

**Published:** 2022-09-13

**Authors:** Ann Corneille Monahan, Sue S Feldman, Tony P Fitzgerald

**Affiliations:** 1 University of Alabama at Birmingham Birmingham, AL United States; 2 Department of Health Services Administration University of Alabama at Birmingham Birmingham, AL United States; 3 School of Mathematical Sciences University College Cork Cork Ireland; 4 School of Public Health University College Cork Cork Ireland

**Keywords:** emergency care, prehospital, emergency, information system, crowding, boarding, exit block, medical informatics, application, health service research, personalized medicine, predictive medicine, model, probabilistic, polynomial model, decision support technique, decision support, evidence-based health care, management information systems, algorithm, machine learning, model, predict, risk

## Abstract

**Background:**

Emergency department crowding continues to threaten patient safety and cause poor patient outcomes. Prior models designed to predict hospital admission have had biases. Predictive models that successfully estimate the probability of patient hospital admission would be useful in reducing or preventing emergency department “boarding” and hospital “exit block” and would reduce emergency department crowding by initiating earlier hospital admission and avoiding protracted bed procurement processes.

**Objective:**

To develop a model to predict imminent adult patient hospital admission from the emergency department early in the patient visit by utilizing existing clinical descriptors (ie, patient biomarkers) that are routinely collected at triage and captured in the hospital’s electronic medical records. Biomarkers are advantageous for modeling due to their early and routine collection at triage; instantaneous availability; standardized definition, measurement, and interpretation; and their freedom from the confines of patient histories (ie, they are not affected by inaccurate patient reports on medical history, unavailable reports, or delayed report retrieval).

**Methods:**

This retrospective cohort study evaluated 1 year of consecutive data events among adult patients admitted to the emergency department and developed an algorithm that predicted which patients would require imminent hospital admission. Eight predictor variables were evaluated for their roles in the outcome of the patient emergency department visit. Logistic regression was used to model the study data.

**Results:**

The 8-predictor model included the following biomarkers: age, systolic blood pressure, diastolic blood pressure, heart rate, respiration rate, temperature, gender, and acuity level. The model used these biomarkers to identify emergency department patients who required hospital admission. Our model performed well, with good agreement between observed and predicted admissions, indicating a well-fitting and well-calibrated model that showed good ability to discriminate between patients who would and would not be admitted.

**Conclusions:**

This prediction model based on primary data identified emergency department patients with an increased risk of hospital admission. This actionable information can be used to improve patient care and hospital operations, especially by reducing emergency department crowding by looking ahead to predict which patients are likely to be admitted following triage, thereby providing needed information to initiate the complex admission and bed assignment processes much earlier in the care continuum.

## Introduction

### Overview

The problem of emergency department (ED) crowding is well known in health care as a complex, multi-dimensional problem that threatens patient safety and care quality and has remained largely unresolved for over 20 years. Despite ED efficiency interventions [1,2] and government policy [3] aimed at reducing crowding, it continues to threaten patient safety and contribute to poor patient outcomes [4-6]. ED crowding occurs when ED demand exceeds the staff’s ability to provide quality care in a reasonable time frame [7,8]. The main causes of crowding are ED “boarding” [9-12] (eg, when an ED bed is occupied by a patient due to be admitted to the hospital, but the patient remains in the ED because no inpatient bed has been assigned) and hospital “exit block” [6] (eg, when patients are delayed or blocked from transitioning out of the ED and into the hospital in a reasonable time frame).

Although some recent literature has attributed ED boarding to insufficient hospital bed capacity [13-17], this description of the situation belies boarding’s complex roots and suggests that hospitals simply do not have inpatient beds available because they are all occupied by patients. In fact, this is rarely the case, as occupancy rates in most US hospitals average 40% for rural hospitals and 65% for urban hospitals [18-20]; these rates have been slowly declining for decades [18-21]. Instead, insufficient bed capacity in most hospitals refers to a shortage of available beds for ED admission. Reasons for this “shortage” include existing bed reservations, which can be for elective surgery patients who might require admission [9,10], for transfer patients from other hospitals, and for geographic bed plans that assign beds to specialties (eg, orthopedics) to keep relevant patients and providers close together [14]. There are positive logistical and care-quality reasons for these bed reservations, but there are also financial reasons that may benefit the hospital yet contribute to ED boarding. For example, reserving beds for highly reimbursable elective procedures that might not be utilized [9], instead of opening the beds for the immediate needs of ED admissions, increases boarding.

Securing hospital beds for ED patients is a time-intensive, interdepartmental negotiation requiring multiple approvals before an ED patient can be transitioned into an inpatient hospital bed. Larger hospitals have bed managers dedicated to effectively utilizing each hospital bed and the patient support services each requires. Much like air traffic controllers, bed managers are the conductors of a complex series of interdependent processes and activities. Bed management involves assessing bed availability throughout the hospital, assessing whether unit resources are in place to enable a particular bed to be filled by a particular patient, identifying additional unit resources that are required to fill a particular bed, determining whether sufficient resources are available to care for specialty patients (eg, cardiac patients) in a general medicine unit, identifying the required resources and available staff (eg, who is at the hospital and who is on call), identifying which beds are reserved for urgent postoperation surgical cases and which are reserved for elective surgeries, and possessing knowledge of matters spanning multiple departments with a multitude of players. This complex and important process may be unnecessarily convoluted in hospitals that have grown in size and responsibility and have become incongruent with effective organizational management. Examinations of clinical workflows for admitting hospital patients from the ED have revealed processes layered with cultural and organizational factors that exacerbate an already inefficient process. There can be 50 to 75 steps between a bed request and time to admission orders, and staff have reported they believe the process is excessively complex, redundant, and in some respects, unsafe [22]. The earlier bed managers have information about a patient who will likely be admitted, the earlier they can begin the bed assignment process, and the earlier the patient can move out of the ED to a hospital bed. This often dysfunctional and protracted hospital transition and bed assignment process blocks patients from transitioning out of the ED to inpatient hospital care (ie, exit block), resulting in the patient waiting long periods of time in the ED for an inpatient hospital bed assignment (ie, boarding).

Boarding negatively affects hospital operations, causing resource strain due to boarded patients’ continued consumption of nurse and physician resources. It precludes the ability to see more patients, because the boarder is occupying an ED bed when an ED level of care is likely not needed [23]. This strain results in a ripple effect throughout the ED that limits all patients’ access to timely emergency care [24] and further impacts the emergency medical services system by increasing ambulance diversion and patient offload time (the time paramedics spend waiting for an ED bed to become available, after which they are able to return to service) [25]. Reduction or removal of the exit block that causes boarding would dramatically reduce the duration of patient boarding. Thus, removal of the 2 main causes of ED crowding (ie, exit block and boarding) would greatly reduce crowding and increase access to care.

Easing ED crowding requires a multifaceted approach. That is to say that there is not one single solution, but rather multiple solutions applied at various points of care that hold promise in easing ED crowding. This paper will report on the use of biomarkers, measured very early in the continuum of care, as a mechanism to predict admission, thereby enabling hospitals to initiate the complex and time-consuming bed management process early and reduce ED crowding.

### Background and Rationale

#### Prior Interventions to Address Boarding and Crowding

Hospitals have implemented a variety of hospital-level and ED-level interventions to reduce boarding and crowding. In terms of hospital-level interventions, some hospitals have reportedly reduced boarding by improving inpatient bed availability, for example by shortening patient stays through better management of hospital services that are not continuously available (eg, catheterizations [26,27]), moving discharged patients awaiting transportation or nonacute care services to “discharge lounges” [28,29], managing discharges in a more timely manner and expediting discharges [30-32], and managing bed cleaning turnaround more efficiently [33-35]. Because ED crowding does not have a singular cause, it also does not have a singular solution. As such, these measures may only address part of the issue; they do not address boarding’s root cause—exit block. Rather, these efficiency measures contribute to process improvement and operational efficiency, because they successfully and sensibly increase hospital bed availability and streamline and simplify processes for providers and administrators, saving them time and improving flow. Thus, they have the potential to reduce boarding and consequent crowding. Hospitals could benefit from early intelligence about demand for beds, and the need to ensure that the right types of beds are available for ED patients.

ED-level interventions have primarily focused on reducing crowding through improvements in ED flow and throughput, such as by using fast tracking [36-38], split-flow processing [39-41], rapid assessment zones [42-44], team triage [45], triage nurse ordering [46], triage standing orders [47], bedside registration [48], physician scribes [49-51], ED flow coordinators [52], point-of-care testing [53-56], and physical expansion of the ED [57]. While some of these measures may make positive contributions to ED efficiency and flow, they are not unlike the hospital-level interventions, which contribute to solving parts of the problem but do not address exit block [58]. Instead, these measures primarily promote efficiency in subsections of the ED care continuum and move patients more quickly toward upstream bottlenecks in the ED process. Even a dedicated ED flow coordinator who successfully increases flow throughout the ED will see much of that benefit lost if improvements are not also implemented outside the ED [52].

#### Interdepartmental Interventions to Address Exit Block

Interventions that have included interdepartmental collaboration with hospital management support have made positive steps toward reducing exit block. Such interventions have resulted in a 68% reduction in the time from inpatient bed requests to receipt of inpatient admission orders (210 minutes to 75 minutes; this does not mean a bed has been assigned, only that the order has been created) and a 25% reduction in the time from inpatient bed requests to patient departure from the ED (360 minutes to 270 minutes) [22]. These interventions are primarily viewed through a process improvement lens, in terms of bed management strategy. For example, a study by Barrett et al [59] reported a 52% reduction in “hold time” (the time from admission decision to departure from the ED) when full-time bed managers were able to identify and assign patients to beds within 15 minutes of a bed request. Another such study, by Howell et al [60], reported a 90-minute reduction between ED patient registration and patient physical departure from the ED for admitted patients when a dedicated “bed traffic controller” was used. The difference between the Barrett et al [59] study and the Howell et al [60] study is that the former employed resources at the micro or patient level, whereas the latter employed resources at the macro or process level, presumably with a top-down view of “traffic.”

The process improvements and bed management strategies reported by Barrett et al [59] and Howell et al [60] also demonstrated how real-time ED data on congestion, flow, and patient admissions can be used by hospital staff outside of the ED, such as the hospital bed manager, to prepare for and manage admissions and bed demand. With reliable information to predict the likeliness of being admitted, effective bed management strategies could be deployed earlier in the admission continuum cycle.

#### Predictive Modeling in Health Care

A variety of models have been used to estimate the risk of hospital admission from the ED, including logistic regression and machine learning, and a variety of predictor variables have been used, including the primary complaint, prior ED visits, referral source, medical history, and mode of arrival. However, model reliance on information that is not readily available (such as patient records) or is inaccurate (such as patient reports of medical history) can be problematic for the application and operation of hospital-admission prediction models. Immediately available point-of-care information from patient biomarkers, such as age, gender, vital signs, and acuity level, offer an advantage over previously collected information [61].

Currently, there is no benchmark to compare hospital admission prediction models. This was evidenced by the authors’ previous systematic review and critical assessment study of models predicting hospital admission, which found that all had potential biases [61].

##### Biomarker Indicators of Admission

The word “biomarker,” short for “biological marker,” refers to a broad category of objective indicators of medical state that can be measured accurately and reproducibly [62]. Examples of biomarkers are age, x-ray images, vital signs, genes, alleles, gender, cognitive state, and acuity level. Vital signs are the most essential biomarker for monitoring hospitalized patients and are the simplest, least expensive, most readily available, and probably the most important information gathered on patients [63]. They are especially useful in the ED environment, which is populated by patients with a variety of symptoms and conditions, challenging care providers to assess patients quickly. Failure to recognize patient severity or acuity can be detrimental or fatal in the ED. Vital signs that are assessed in real time provide an opportunity to avert this risk to patients, because changes in vital signs have been shown to occur several hours before serious adverse events [64-68]. As such, vital signs can be used to identify ED patients at risk of deterioration [67-72].

The purpose of this study was to report on the development of a model that used patient biomarkers collected at triage (the 5 vital signs and age, gender, and acuity level) for the early prediction of the risk of imminent hospital admission or transfer from the ED for adult patients.

## Methods

### Study Design

This retrospective cohort study evaluated 1 year of consecutive data events for adult patients admitted to the ED and developed an algorithm to predict which patients would require imminent hospital admission. Eight variables collected at triage were evaluated for their role in the outcome of the patient ED visit. Logistic regression was used to model the study data.

### Study Setting, Data Source, and Population

The sample population of deidentified data was drawn from 1 year (January 1, 2019, through December 31, 2019) of consecutive ED admissions to an academic medical center and were queried from its Informatics for Integrating Biology to the Bedside (i2b2) database [73], part of the National Institutes of Health–funded National Centers for Biomedical Computing. Transfer patients (ie, ED patients requiring inpatient hospital admission who were transferred to other hospitals for clinical reasons, such as to receive specialty care) were grouped with admitted patients, because their clinical presentation and reasons for transfer to other facilities for inpatient admission were clinically identical to those of admitted patients [74]. The academic medical center ED is a 48-bed, level-1 adult trauma center with an average daily ED census of 300 patients. The hospital has 1157 beds enterprise-wide.

All clinical data were collected at triage by nurses and entered into the electronic medical record at the point of care. Standardized methods were used to collect vital signs and acuity level.

#### Inclusion and Exclusion Criteria

All adult (ie, age ≥18 years), nonpsychiatric, nonobstetric, fully triaged patients admitted to the ED (including those transferred to other hospitals for admission) and subsequently admitted to the hospital or discharged from the ED were included in this study. Psychiatric, pediatric (ie, age <18 years), and obstetric patients were excluded. Psychiatric and obstetric patients were excluded because these populations’ symptomology, and the clinical variables that are evaluated to determine their course of treatment, are significantly clinically different from the general-medicine population [75,76]. Pediatric patients were excluded because the threshold for admission for these patients is lower than for adults [77], and their inclusion would result in overly sensitive inclusion criteria for adults.

#### Selection of Variables for Measurement

The choice of variables evaluated (Table 1) for model development was derived from a systematic review of studies evaluating models designed to predict hospital admission, which suggested variables that were most valuable for patient admission or discharge [61]. Predictors were selected a priori by expert knowledge. Although the literature suggests that SpO_2_ [78], level of consciousness [79], and mode of arrival [78,80-85] are important variables for consideration [61], the available data were too inconsistent and, therefore, were not included in this model.

**Table 1:** Eight variables were analyzed for their utility in predicting hospital admission and discharge.

Variables				Means of collection
**Predictor variables**				
	Age			Provided by patient (or family or friend if patient was unable to report)
	Acuity			The standardized 5-level Emergency Severity Index [86] was used by the triage nurse to categorize patient acuity from most urgent (level 1) to least urgent (level 5)
		Systolic blood pressure; diastolic blood pressure; heart rate, respiration rate; temperature	Typical, standardized methods were used to collect vital signs; blood pressure was the only variable collected by 2 methods: manual (the primary method) and automated	
	Gender			Provided by patient (or family or friend if patient was unable to report); if the patient was unaccompanied, clinicians determined gender by visual inspection
**Outcome variable**				
	Admitted or discharged			Determined by physician

### Study Protocol and Data Management

The data were exported from i2b2, imported into an Excel table for review and cleaning, then exported to the Stata statistical package (version 14.1; StataCorp) for analysis. The data were evaluated for missing values.

### Data Analysis and Model Development

Data distribution was investigated with summary statistics and histograms. We examined the univariate associations of age, systolic blood pressure (BP), diastolic BP, heart rate, acuity, and gender with the probability of admission using a logistic regression. Traditional logistic regression assumes that the association between continuous risk factors and the probability of admission is linear on a log-odds scale. We considered a more flexible model in which continuous risk factors were included as fractional polynomials, a model-building technique that allows for nonlinear associations [87], and temperature, respiration, and acuity were included as categorical risk factors.

We performed a multivariable fractional polynomial (MFP) analysis that included all risk factors. Temperature values were tightly clustered, with 97% of values between 36.1 °C and 37.8 °C, with a wide spread in values above and below these values. For the purposes of modeling, we created a modified temperature variable where values less than 36.1 °C or greater than 37.8 °C were truncated. Fractional polynomials of the modified temperature were combined with dummy variables indicating high (>37.8 °C) and low (<36.1 °C) temperatures. Respiration was included as a categorical variable due to difficulty in modeling the association between respiration as a continuous variable and the probability of admission. The MFP analysis included nonlinear relationships if they were sufficiently supported by the data. The fit of a second order fractional polynomial was compared to that of the null model, the linear model, and finally to the optimal first-order polynomial. Convergence was achieved when the functional forms did not change. The significance level for the comparison of fractional polynomial models was set equal to 0.01. As some subjects visited the ED more than once, we considered a robust MFP analysis that allowed for correlation between repeat observations of the same subject. Descriptive statistics considered each patient visit as unique. Patient numbers refer to the number of ED encounters.

Model performance was assessed by discrimination and calibration. Discrimination, the model’s ability to accurately distinguish between admission and nonadmission [88], was measured with the area under the receiver operating characteristics curve (AUROC) [89]. To assess potential overoptimism, we also calculated a 10-fold cross-validated AUROC. Calibration, the extent to which the model-predicted probabilities agree with observed binary outcomes [90], is a more appropriate gauge of model performance [91] and was measured by Hosmer-Lemeshow goodness of fit and evaluated graphically using a “calibration belt” [92] for internal validation. The calibration belt methodology formulated the relationship between the predictions and the true probabilities of admission with a second logit regression model based on a polynomial transformation of the predictions. The degree of the polynomial was forwardly selected, beginning with the second order on the basis of a sequence of likelihood-ratio tests [91].

The model was designed to be hospital-specific with application to a particular ED population. As such, we did not measure external validity. Risk factors were evaluated for extreme values, resulting in the loss of less than 2% of patient events overall.

### Ethical Considerations

Ethical approval to conduct the investigation was obtained from The University of Alabama at Birmingham Institutional Review Board (IRB-300007437). This study was conducted on a data set that was void of any protected health information.

## Results

### Descriptive Data

The population consisted of 93,847 adults (age ≥18 years) who were fully triaged, general-medicine (ie, nonpsychiatric and nonobstetric) patients admitted to the ED from January 1, 2019, through December 31, 2019, and subsequently discharged from the ED or admitted to the hospital. The mean age of the 93,847 patients was 46.3 years; 55.6% (52,147) were female; 56.4% (52,974) had acuity level 3; mean systolic BP was 139 mmHg; mean diastolic BP was 84 mmHg; mean heart rate was 87.2 beats/minute; mean respiration rate was 17.7 breaths/minute; and mean temperature was 36.8 °C (Table 2). Temperature and respiration rate had long-tailed, tightly clustered distributions. Temperature ranged from 27 °C to 40.3 °C with only 1% (938) of values less than 36 °C and 1% (938) greater than 38.4 °C. Respiration rate was recorded as a whole number and was clustered at even numbers, with 26% (24,400), 40% (37,539), and 12% (11,262) of subjects having respiration rates of 16, 18, and 20 breaths per minute, respectively, ranging from 10 to 40, with 1% (938) of values less than 14 and 1% (938) greater than 26. Compared to those not admitted, admitted patients were more likely to be male; be older; have lower systolic BP and lower diastolic BP; have higher heart rate, respiration rate, and temperature; and be more acute, as indicated by a lower Emergency Severity Index (ESI) [86] level. This index has a scale of 1 to 5, with 1 being most urgent and 5 being least urgent. Of those admitted, 45% (5779/12,711) had acuity scores less than 3, compared to only 8% (6426/81,136) of those not admitted.

**Table 2:** Values for predictor variables by admission status.

Variables		Not admitted (N=81,136)	Admitted (N=12,711)	Total (N=93,847)
Age (years), mean (SD)		44.8 (17.4)	55.8 (16.9)	46.3 (17.3)
**Gender, n (%)**				
	Male	35,169 (43.3)	6531 (51.4)	41,700 (44.4)
	Female	45,967 (56.7)	6180 (48.6)	52,147 (55.6)
Systolic blood pressure (mm Hg), mean (SD)		139.1 (23.2)	137.9 (28.5)	139.0 (50)
Diastolic blood pressure (mm Hg), mean (SD)		84.4 (13.5)	81.2 (16)	84. (13.9)
Heart rate (beats/minute), mean (SD)		86.3 (15.5)	92.9 (18.6)	87.2 (16.1)
Respiration rate (breaths/minute), mean (SD)		17.6 (1.8)	18.6 (3.1)	17.7 (2.1)
Temperature (°C), mean (SD)		36.8 (0.4)	36.8 (0.6)	36.8 (0.4)
**Emergency Severity Index level, n (%)^a^**				
	1	23 (0)	571 (4.5)	594 (0.6)
	2	6403 (7.9)	5208 (41)	11,611 (12.4)
	3	46,454 (57.3)	6520 (51.3)	52,974 (56.4)
	4	26,128 (32.2)	385 (3)	26,513 (28.3)
	5	2128 (2.6)	27 (0.2)	2155 (2.3)

^a^Ranges from most urgent (1) to least urgent (5).

### Probability of Admission

**Figure 1:**
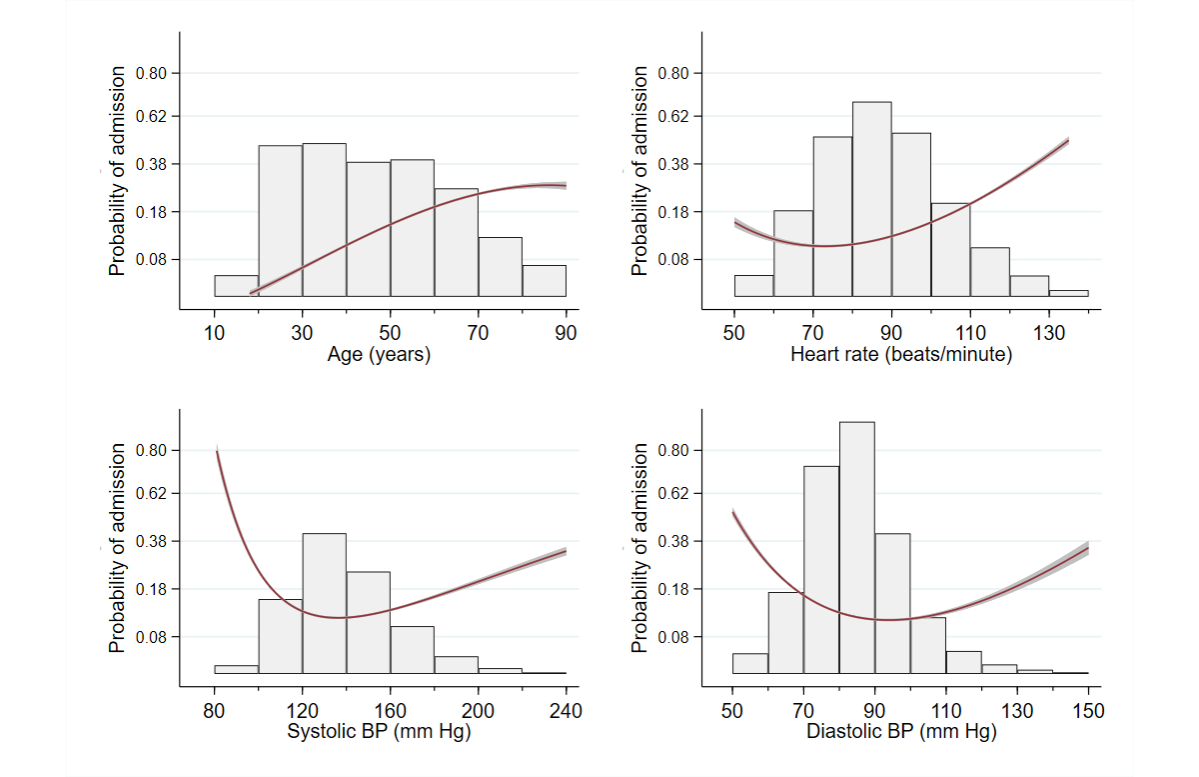
Histograms of variable distributions overlaid with graphs showing the probability of admission on a log-odds scale. The shaded areas around the curves represent the 95% CI. BP: blood pressure.

**Figure 2:**
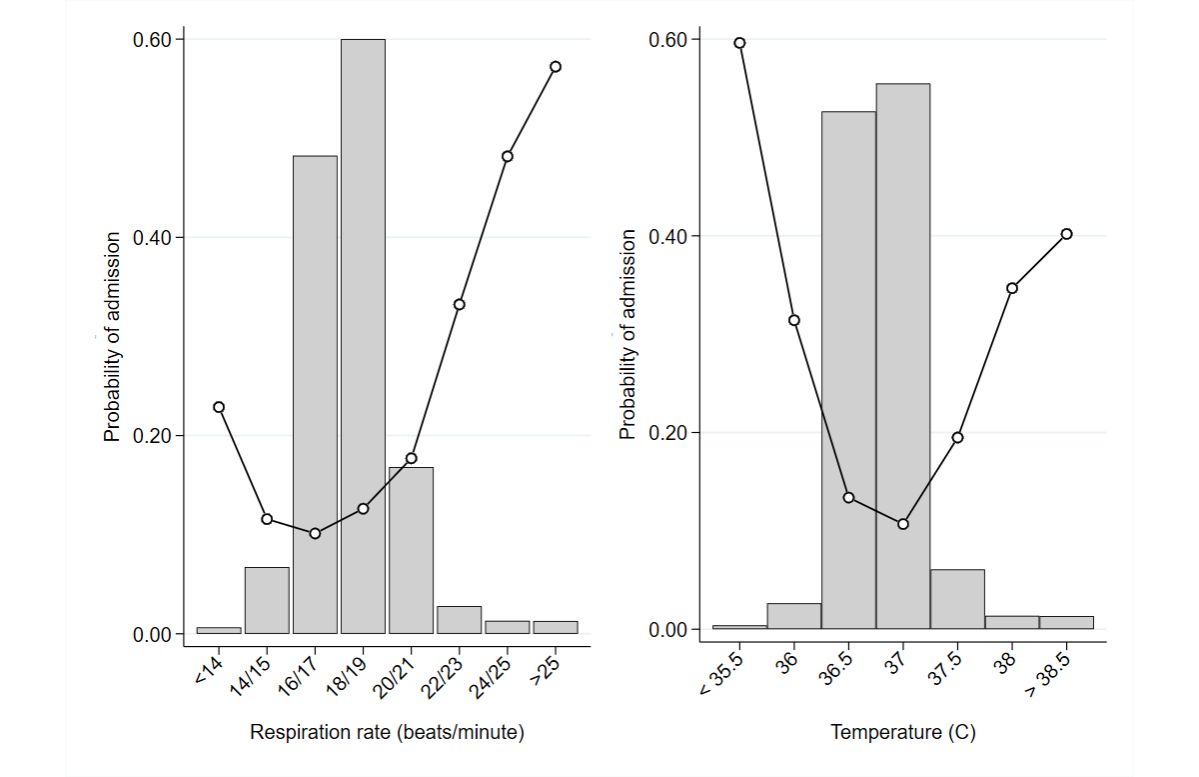
Histograms of variable distributions overlaid with graphs showing the observed admission rates.

Male admission rates were higher, at 15.7% (6531/41,700) compared to 11.9% (6180/52,147) for women. There was a strong association between acuity and admission, with the probability of admission falling sharply from 96.1% (571/594) for the most urgent patients (ESI 1) to 1.1% (23/2155) for the least urgent patients (ESI 5) (Table 3). Figure 1 shows variable distributions and their associations with the probability of admission, shown on a logit scale. Results are based on second order fractional polynomials. There was a clear, nonlinear association for all continuous variables except age. The probability of admission increased until age 80 and then leveled off. For systolic BP, diastolic BP, heart rate (Figure 1), respiration rate, and temperature (Figure 2), the association was nonlinear, with the probability of admission lowest at the center of the distribution and higher at the extremes. For example, for systolic BP, the probability of admission was lowest, at 15%, for values between 120 mm Hg and 150 mm Hg, and the probability of admission continued to rise at values outside this range, reaching 95% for values <75 mm Hg and a probability of 40% at values >235 mm Hg. Similarly, at a temperature of 36.7 °C, the probability of admission was lowest, at 17%, increasing to 50% at 39.4 °C and to 80% at 35 °C.

The MFP logistic regression showed significant associations with acuity, respiration, and gender, first-order fractional polynomials for age, and second-order fractional polynomials for systolic BP, diastolic BP, temperature, and heart rate.

**Table 3:** Probability of hospital admission by acuity level. The most acute patients are at Emergency Severity Index levels 1 and 2, with the least urgent at level 5. A total of 12,711 of 93,847 (13.5%) patients were admitted.

Emergency Severity Index level	Total patients, n	Admitted patients, n	Probability of admission, %
1	594	571	96.1
2	11,611	5208	44.9
3	52,974	6520	12.3
4	26,513	385	1.5
5	2155	27	1.1

### Fit and Calibration

**Figure 3:**
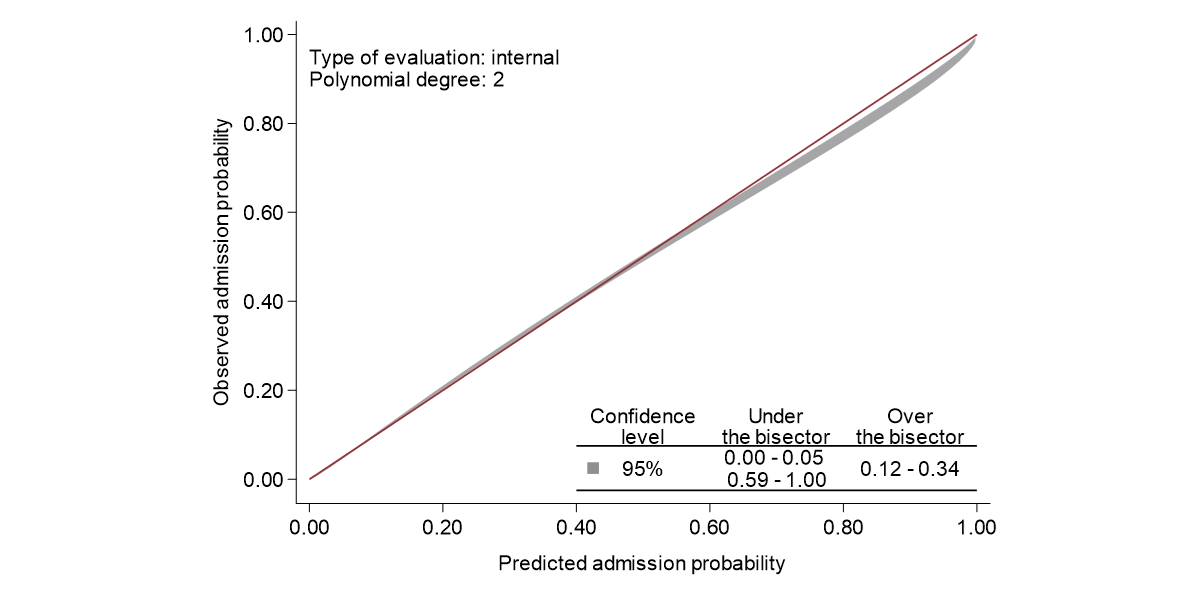
Calibration belt of observed versus predicted admission probabilities. The bisector is the line of perfect calibration. The calibration belt (shown in gray) represents the 95% confidence level calibration of the model.

In Figure 3, the observed admission rate is plotted against the predicted admission rate based on the results of our final logistic regression model. There was excellent agreement between observed probabilities and predicted probabilities based on the model. The 95% CI fell below the identity line at the high end, which indicated that the model slightly overpredicted risk for patients who had a probability of admission over 0.59. This difference in probabilities was less than 0.04. For admission probabilities less than 0.59, the bias was less than 0.01.

### Model Discrimination

**Figure 4:**
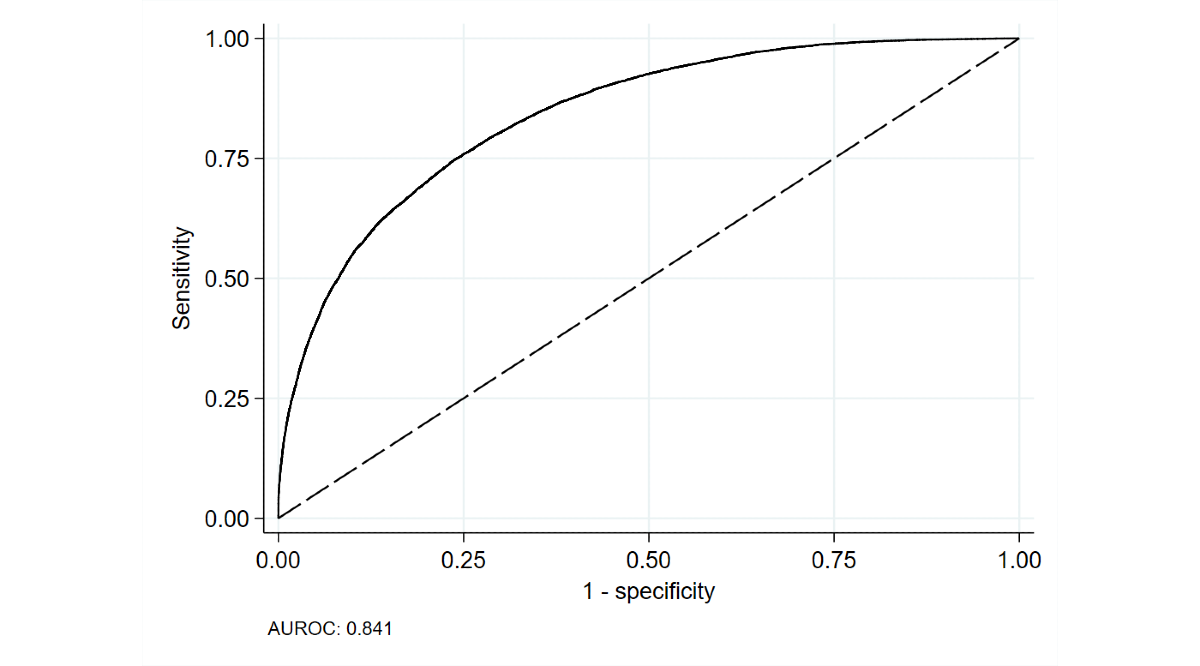
Area under the receiver operating characteristics curve for predicting admission. AUROC: Area under the receiver operating characteristics curve.

Model discrimination was measured with the AUROC (Figure 4). The AUROC was 0.841, indicating that the model had good ability to discriminate between patients who would and would not be admitted [93]. The 10-fold cross-validated AUROCs ranged from 0.839 to 0.842.

### Missing Values

Age and gender values were complete for all 113,739 patients. Missing values for all other variables were very low, ranging from 1.5% to 1.9% (1650 to 2143), except for temperature and acuity, which were missing for 6.8% (7685) and 9.2% (10,419) of patients, respectively. As 83% (8,647/10,419) of cases missing acuity had been admitted, this group was identified as “missing not at random.” We found no evidence to suggest that temperature was not “missing at random,” and the degree of missingness was low enough that we did not expect it to bias results. Cases with missing values were excluded.

## Discussion

### Principal Findings

We have illustrated the application of sophisticated fractional polynomials to identify and model nonlinear associations between risk factors and the probability of admission using immediately available patient biomarkers (age, systolic BP, diastolic BP, heart rate, respiration rate, temperature, gender, and acuity level) collected at triage. The resulting prediction model exhibited excellent calibration with good agreement between observed and predicted admissions at all risk-of-admission levels. The model showed good ability to discriminate between patients who would and would not be admitted. Methodological techniques promoted internal validity and mitigated against overfitting and endogeneity, which can arise when predictor variables are correlated with the outcome due to their relationship with variables not in the model [94]. Given our large sample size, a priori inclusion of risk factors, and predictor selection based on topic knowledge, the risk of variable omissions was reduced, and the risk of overfitting due to the use of optimal fractional polynomials was not a concern. A 10-fold cross-validation yielded almost identical AUROC values.

Categorization of respiration rate and truncation of temperature could cause loss of relevant information [95]. However, our transformations were informed by the data: our cut-off points for respiration rate reflected how it was recorded (ie, responses were usually 1 of 3 values and showed a preference for even numbers), and although temperature was truncated, less than 4% (3753/93,847) of observations were affected, indicator variables for low and high temperature were included, and temperature was included as a continuous variable.

Anecdotal information suggests that in the practice of a busy ED, failure to record information deemed nonessential can occur when a patient is already scheduled for hospital admission, and this is most likely to occur when the patient is receiving life-saving care. These cases are very acute and are likely to be admitted. The patients with a missing acuity level tended to be very acute (ie, most cases with missing acuity were admitted) and we were comfortable excluding them, because they were not the patient group that the model aimed to identify as requiring admission; these patients likely had already been identified as urgently needing care and were already likely to be admitted. This is a site-specific model designed to operate in a test environment and show proof of concept; generalizability is not assumed. Because this model uses standardized biomarker data and not data specific to the study environment, it is possible that with recalibration, this model could be useful outside the study environment. It is worth noting, however, that in addition to the real-time availability of electronic patient data, a requirement of model implementation is an application to retrieve the data and apply it to the model algorithm to produce a patient’s likelihood of admission, then provide the information to bed managers to begin securing patient beds early.

This model, as proposed, has real-world utility for those involved in the patient admission continuum, because it allows patients to be moved out of the ED sooner, thereby easing exit block and benefiting patient care and hospital operations [59,60]. The model’s reliance on biomarkers that are routinely collected at the initial point of care (ie, ED triage) and have standardized definitions, measurements, and interpretations [62] is advantageous for a model that can be used very early in the patient care continuum. That, however, does not imply that model development and implementation within a setting is easy. Rather, the data coming from electronic medical records may require labor-intensive preparation to make it suitable for model development and implementation.

This model also showed that a hospital can develop a system for identification of patients at high risk of admission for use in resolving problems such as exit block. The model can be adapted to other ED environments using each ED’s individual data.

### Comparison With Prior Work

Addressing ED overcrowding and exit block cannot be accomplished by applying a one-size-fits-all solution. The most recent prior work in this area centers around different methods and models for addressing the same problem—ED crowding. For example, Acuna et al [96] optimized ED crowding by creating an ambulance allocation model that led to a 31% improvement in ED crowding, and Isfahani et al [97] used a computer simulation model to assess the effect of ED discharge lounges, finding there was a 5% reduction in admission waiting times. In terms of applying algorithms, Brink et al [98] developed an 8-variable model to predict hospital admission for elderly patients and Marcusson et al [99] developed a 38-variable model to predict hospital admission for elderly patients; both models aimed at helping patients receive care sooner.

### Limitations

The applicability of this study should be understood in the context of its limitations. As mentioned earlier, this study was performed as a proof of concept in a large academic medical center and may lack generalizability to other environments. If this model were applied in a setting where complex processes to secure inpatient beds are not undertaken by the hospital (involving, for example, providers, equipment, and other specialized resources for different patient conditions), or where securing beds does not require a large amount of time, then the time-saving advantages of this model would not be realized by bed managers. Additionally, there may have been confounding factors that were mediating or moderating factors in our model and outside the scope of this study. Lastly, while exit block and ED boarding have been reported internationally, this study was conducted in a US hospital, and is not internationally generalizable. Perhaps a similar, recalibrated version of our derived model would have applications in divergent ED settings. However, we recommend that other hospitals develop hospital-specific models using the MFP modeling techniques presented here.

### Conclusion

This primary data study illustrates the application of a site-specific risk prediction model to reduce ED crowding due to exit block. MFPs were used to predict the probability of admission based on 8 biomarkers (5 vital signs and age, gender, and acuity level) and to generate variables utilized by the logistic regression model to produce a site-specific formula to analyze future input data. This intervention can reduce ED exit block, the known source of ED boarding and crowding, by enabling the hospital to seek and requisition hospital beds earlier and transition ED patients into those beds earlier. This intervention requires interdepartmental collaboration with the support of hospital management to be successfully implemented into hospital structures and processes. Compared to other interventions in the hospital admission and bed assignment process that have successfully reduced crowding [22,59,60], this model goes a step further by looking ahead to predict which patients will be admitted, thereby providing the needed information to initiate admission and bed assignment processes much earlier in the care continuum. The model’s prediction of patient admissions combined with the utility of real-time hospital data to improve congestion, flow, and patient admissions [59,60] results in a powerful tool to impact the ED crowding crisis.

## Abbreviations

AUROC: area under the receiver operating characteristics curve
BP: blood pressure
ED: emergency department
ESI: Emergency Severity Index
MFP: multivariable fractional polynomial
